# The profile of patients diagnosed with Parkinson’s disease at the Charlotte Maxeke Johannesburg Academic Hospital

**DOI:** 10.4102/jcmsa.v4i1.425

**Published:** 2026-10-08

**Authors:** Itumeleng I. Lamola, Demetri G.A. Veliotes, Andre Mochan

**Affiliations:** 1 Division of Neurology, Department of Neurosciences, School of Clinical Medicine, Faculty of Health Sciences, University of the Witwatersrand, Johannesburg, South Africa; 2 Department of Physiology, Faculty of Health Sciences, University of the Witwatersrand, Johannesburg, South Africa

**Keywords:** idiopathic Parkinson’s disease, demographic profile, UPDRS, Hoehn and Yahr, South Africa

## Abstract

**Background:**

Idiopathic Parkinson’s disease (IPD) is the second most common neurodegenerative disorder after Alzheimer’s disease, but data from Sub-Saharan Africa are scarce. This study describes the demographic and clinical characteristics of IPD patients at the Charlotte Maxeke Johannesburg Academic Hospital movement disorder clinic and evaluates disease severity with the Unified Parkinson’s Disease Rating Scale (UPDRS-III) and the Hoehn and Yahr staging scales.

**Methods:**

A prospective cohort study was conducted from May 2024 to May 2025, enrolling adult patients (≥ 18 years) with a diagnosis of IPD.

**Results:**

Eighty-two patients were included (mean age 68.2 years, s.d. 10.9; 59.8% male; 52.4% Black African). Median disease duration was 3 years (interquartile range [IQR] 1–6). Levodopa monotherapy was the most common treatment (42.7%), followed by combinations with amantadine or pramipexole. The median UPDRS-III score was 60 (IQR 48–76), with 73.2% classified as having severe disease. The Hoehn and Yahr staging revealed that 39% of patients were at Stage 4 and 11% at Stage 5.

**Conclusion:**

Most patients in this cohort had advanced disease at presentation, underscoring the need for earlier diagnosis and timely referral to specialist clinics.

**Contribution:**

These findings highlight the importance of strengthening movement disorder services in South Africa and suggest that delayed presentation may contribute to the high burden of severe disease in this population.

## Introduction

The hallmark pathological feature of idiopathic Parkinson’s disease (IPD) is progressive degeneration of dopaminergic neurons in the substantia nigra pars compacta, resulting in reduced dopamine transmission within the basal ganglia circuitry.^1^ Disruption of this dopamine signalling leads to the cardinal motor features of IPD, including bradykinesia, rigidity, tremor, and postural instability. Additionally, the presence of Lewy bodies, composed of misfolded α-synuclein, contributes to both motor and non-motor manifestations of IPD, including cognitive impairment, sleep disturbances, and autonomic dysfunction.^2^ The pathogenesis of IPD reflects complex interactions between genetic factors, environmental exposures, and abnormal protein aggregation.

In low- and middle- income countries (LMICs) such as South Africa, diagnosis often relies entirely on clinical criteria, requiring the presence of bradykinesia with either rigidity or resting tremor and supported by a sustained response to dopaminergic therapy.^1,3^ Poor health awareness and education are pervasive, and patients may present late due to limited awareness, poor health-seeking behaviour, and restricted access to tertiary services. As a result, diagnosis may often occur at more advanced stages, reducing patients’ quality of life by delaying diagnosis and treatment and complicating long-term management. Studies on the prevalence of IPD have been predominantly conducted in high-income countries (HICs), creating a gap in our understanding of its impact in LMICs.^4^

Idiopathic Parkinson’s disease is a progressive disorder, with motor and non-motor manifestations worsening over time.^1^ While early stages may be dominated by non-motor features like constipation, anosmia, and rapid eye movement (REM) sleep behaviour disorders followed by motor symptoms of tremor and bradykinesia, later disease is characterised by gait freezing, postural instability, motor fluctuations, and cognitive decline. Clinicians can monitor progression through neurological assessment and examination. However, the severity and progression of IPD are better assessed and monitored using structured tools like the Hoehn and Yahr scale, the Unified Parkinson’s Disease Rating Scale (UPDRS), and Movement Disorder Society-Unified Parkinson’s Disease (MDS-UPDRS), and quality-of-life measures such as the Parkinson’s Disease Questionnaire-39 items (PDQ-39).^5^

### Treatment of idiopathic Parkinson’s disease

Pharmacological therapy remains the cornerstone of management, with levodopa the most effective agent for alleviating motor symptoms.^6^ Adjunctive therapies include dopamine agonists and monoamine oxidase-B (MAO-B) inhibitors, which are used to improve motor fluctuations or delay the initiation of levodopa. For patients with advanced or late treatment-refractory disease, device-aided therapies such as levodopa-carbidopa intestinal gel infusion, apomorphine pumps, and deep brain stimulation (DBS) provide further options.^1^ However, no current therapy alters disease progression, and treatment is therefore aimed at optimising symptom control and quality of life.

In LMICs, treatment options are often limited by poor drug availability, high costs, and restricted access to movement disorder specialists.^7^ Levodopa-carbidopa monotherapy is frequently the only accessible therapy, while dopamine agonists and advanced interventions such as DBS are largely unavailable.^8^ This reliance on basic therapy, coupled with late referral, results in patients often presenting with more advanced disease as compared with HICs.

### Context in South Africa and study rationale

As life expectancy increases in Sub-Saharan Africa, neurodegenerative diseases such as IPD are becoming a significant public health concern.^7^ However, there remains little epidemiological data from this region, and the clinical profile of patients with IPD in South Africa is poorly described. In resource-constrained tertiary hospitals such as Charlotte Maxeke Johannesburg Academic Hospital (CMJAH), patients often present late in the disease course, with more advanced disease, and have fewer treatment options than counterparts in HICs. These differences in diagnosis timelines, severity at presentation, and treatment access highlight the importance of local data to guide care and resource allocation. This study aimed to describe the demographic and clinical characteristics of patients with IPD attending the CMJAH movement disorder clinic, focusing on disease severity as measured by the UPDRS-III and the Hoehn and Yahr staging.

## Research methods and design

### Study design

This study was a prospective cohort study, based on chart review, patient interviews, and clinical examination of outpatients diagnosed with IPD attending the movement disorder clinic at CMJAH.

### Study setting

Patients who were diagnosed with IPD, on treatment, and attending the movement disorder clinic at CMJAH were included.

### Data collection

Patients with IPD were identified in the movement disorder clinic register at the CMJAH neurology clinic. Identified patients had their medical records reviewed to confirm diagnosis and treatment. A data collection tool was used to systematically review the medical records of patients. Information on demographic characteristics (age, biological sex, race) and date and year of diagnosis was extracted from the records.

Prospective assessment of disease motor severity was determined using the MDS-UPDRS3 measurement scale, with permission obtained from the Movement Disorder Society (MDS). This scale measures motor function across speech, facial expression, rigidity, finger tapping, hand movements, pronation or supination of hands, toe tapping, leg agility, rising from chair, gait, freezing of gait, and postural stability. This score was reported as a median and interquartile range (IQR) and summarised according to age, sex, race, treatment regimen, and duration of treatment. The Hoehn and Yahr Stage is a five-level clinical staging system that is widely used to assess disease severity prospectively based on information from the motor assessments.

### Data analysis

Data collected were captured in a password-protected study-specific Excel spreadsheet (Microsoft Corporation, Seattle, WA, United States). Data from the Excel spreadsheet were exported into Stata version 18.5 (Stata Corporation, College Station, TX, United States) for analysis. Descriptive statistics described the patients included in the study. Means and standard deviations were used to describe patients with respect to continuous variables that were normally distributed, while medians and IQRs were used to describe patients with respect to variables that were non-normally distributed. Distributional plots and the Shapiro-Wilk test were used to determine normality of continuous variables. Frequencies and percentages were used to describe the patients with respect to categorical variables. The Rank-sum test was used to test the hypothesis that the UPDRS3 score differed by age categories, sex, race and treatment regimen, while the Chi-square test or Fisher’s exact test was used to test the hypothesis that severe diseases differed by age categories, sex, race and treatment regimen.

### Ethical considerations

Ethical clearance was obtained from the University of the Witwatersrand Research Ethics Committee (Clearance no: M231123). Unique study numbers were randomly allocated to participants to maintain anonymity. Permission from the Chief Executive Officer (CEO) of CMJAH and the Gauteng Department of Health was obtained to conduct the study in the facility.

## Results

### Socio-demographic characteristics

A total of 82 participants were included in the analysis. This sample size achieved 98.7% power to detect the observed prevalence of severe disease (73% ± 10%) at a significance level of 0.05. The mean age of participants was 68.2 years (s.d. 10.9; range 40–91 years). The largest proportion fell within the 70–79-year age group (41.5%), followed by those aged 60–69 years (25.6%). Only 6.1% of participants were younger than 50 years.

Males comprised most of the cohort (59.8%). More than half of the participants were black African people (52.4%), followed by white people (32.9%), Indian people (12.2%), and mixed race people (2.4%). Table 1 summarises patient characteristics.

**TABLE 1:** Demographic characteristics.

Variable	*n*	%	Mean	s.d.
**Age in years**	-	-	68.2	10.9
**Age categories**				
40–49	5	6.1	-	-
50–59	13	15.6	-	-
60–69	21	25.6	-	-
70–79	34	41.5	-	-
80+	9	11.0	-	-
**Biological sex**				
Female	33	40.2	-	-
Male	49	59.8	-	-
**Race and/or ethnicity**				
Black African people	43	52.4	-	-
White people	27	32.9	-	-
Indian people	10	12.2	-	-
Mixed race people	2	2.4	-	-

Note: Socio-demographic characteristics of the 82 patients with idiopathic Parkinson’s disease enrolled at Charlotte Maxeke Johannesburg Academic Hospital.

IPD, idiopathic Parkinson’s disease; CMJAH, Charlotte Maxeke Johannesburg Academic Hospital; s.d., standard deviation.

### Clinical profile and treatment patterns

Levodopa-based therapy was used in all patients, with levodopa plus amantadine being the most frequent regimen followed by levodopa monotherapy (Figure 1).

**FIGURE 1:**
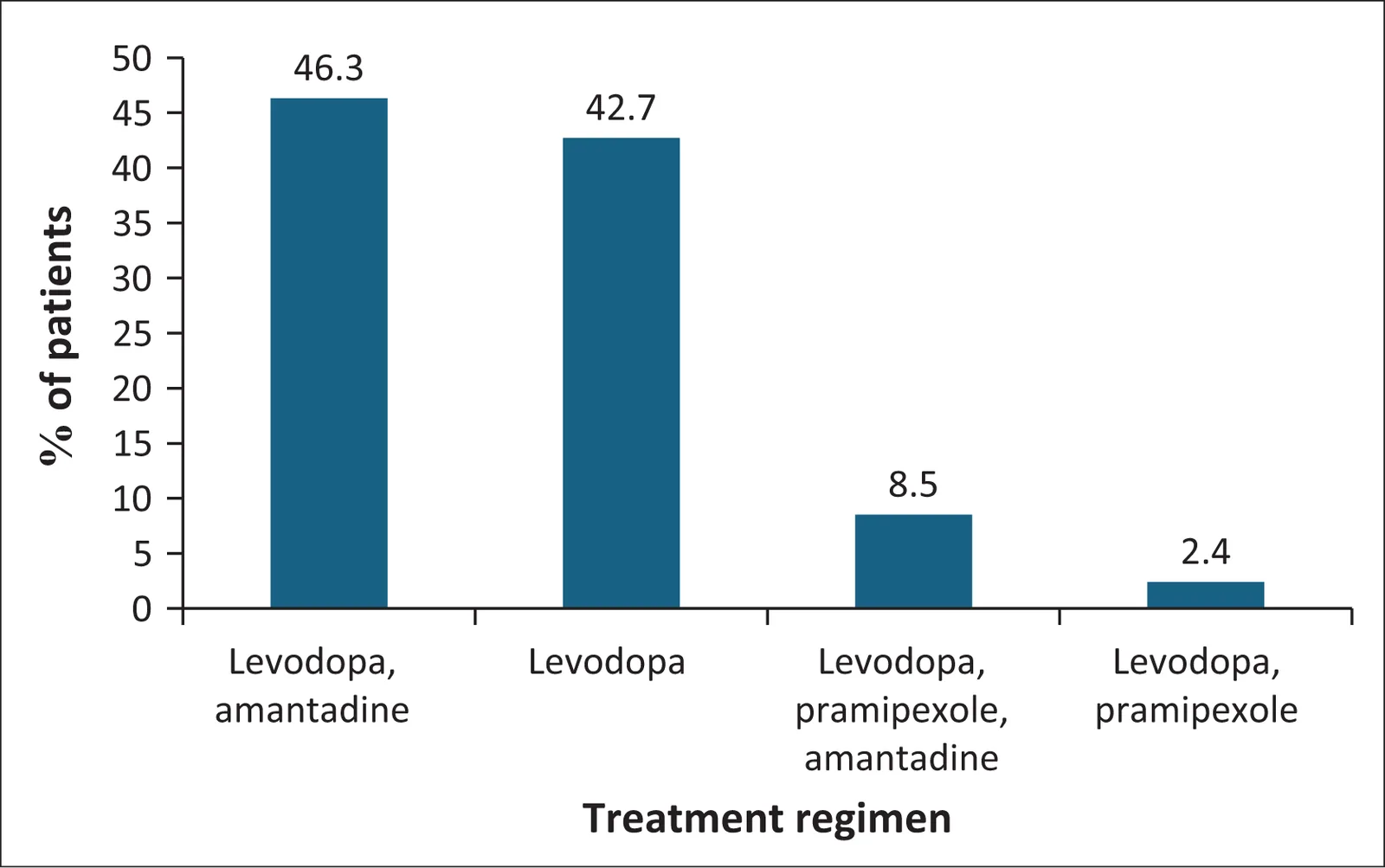
Treatment regimens taken by patients with idiopathic Parkinson’s disease at the movement disorder clinic at the Charlotte Maxeke Johannesburg Academic Hospital (*N* = 82).

### Motor severity: Unified Parkinson’s Disease Rating Scale-III

Motor severity was assessed using the UPDRS-III, which ranges from 0 to 132. A score > 59 is classified as severe disease, 33–58 as moderate, and ≤ 32 as mild. The cohort had a median UPDRS-III score of 60 (IQR 48–76; mean 61.0, s.d. 19.2), placing most participants in the severe range (Figure 2).

**FIGURE 2:**
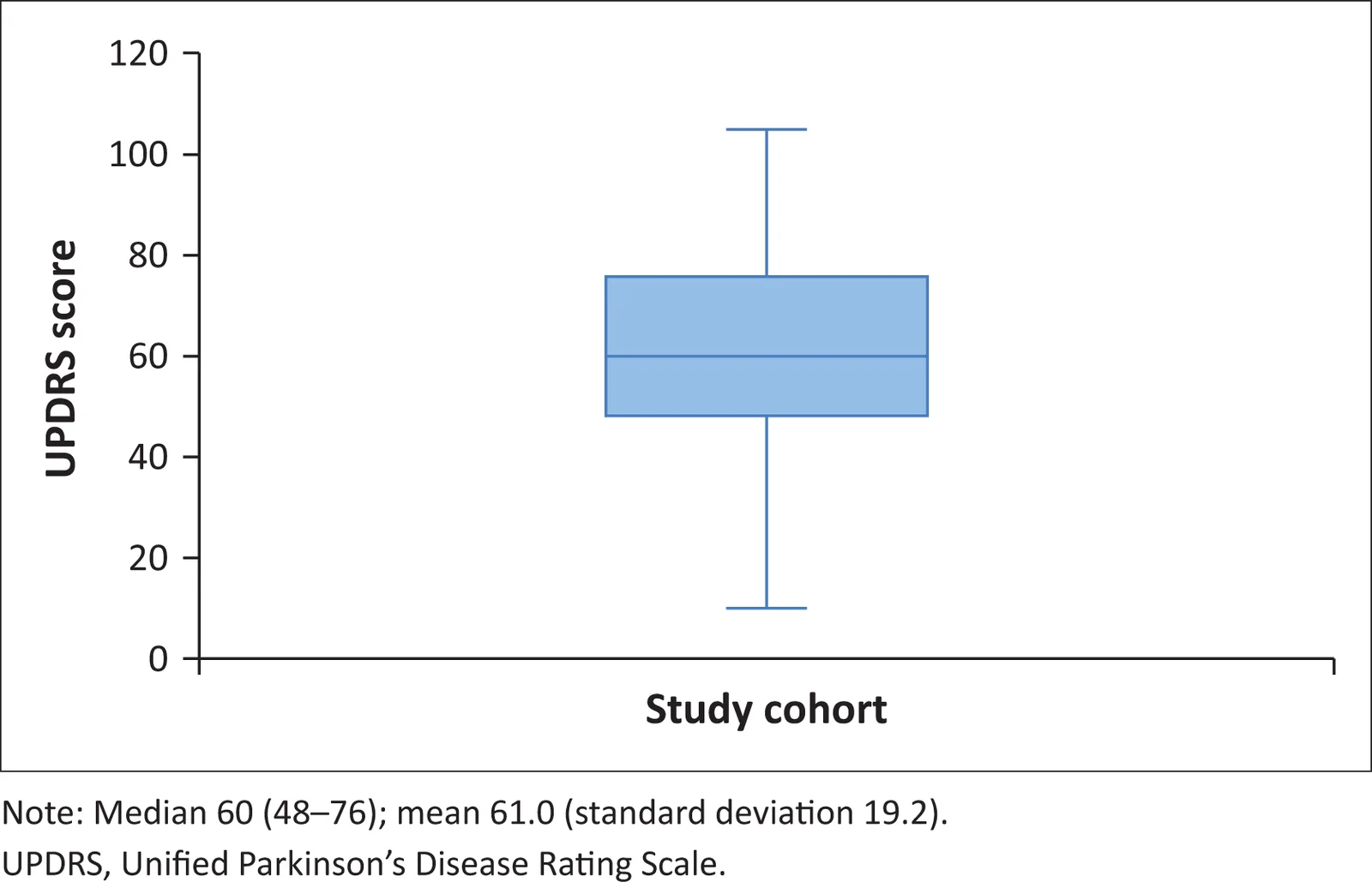
Box plot of Unified Parkinson’s Disease Rating Scale scores.

Overall, 60 of 82 participants (73.2%) met criteria for severe motor disease. Among those with severe disease, the median UPDRS-III score was 67 (IQR 41–105), compared with 44 (IQR 10–58) among those with mild-to-moderate disease.

### Motor severity: Hoehn and Yahr staging

Disease severity was also evaluated using the Hoehn and Yahr staging system. Most patients were classified as Stage 4 (53.3% of those with severe disease on UPDRS), consistent with severe disability but retained the ability to stand and walk unassisted as per Hoehn and Yahr staging. Nine patients with severe disease were in Stage 5, indicating complete dependence.

Among participants with mild-to-moderate disease, stages ranged from 1 to 2.5, with 50% falling into Stage 2.5 (bilateral disease with mild postural instability). This distribution aligned closely with UPDRS-III scores, reinforcing the high burden of advanced disease in the cohort (Figure 3).

**FIGURE 3:**
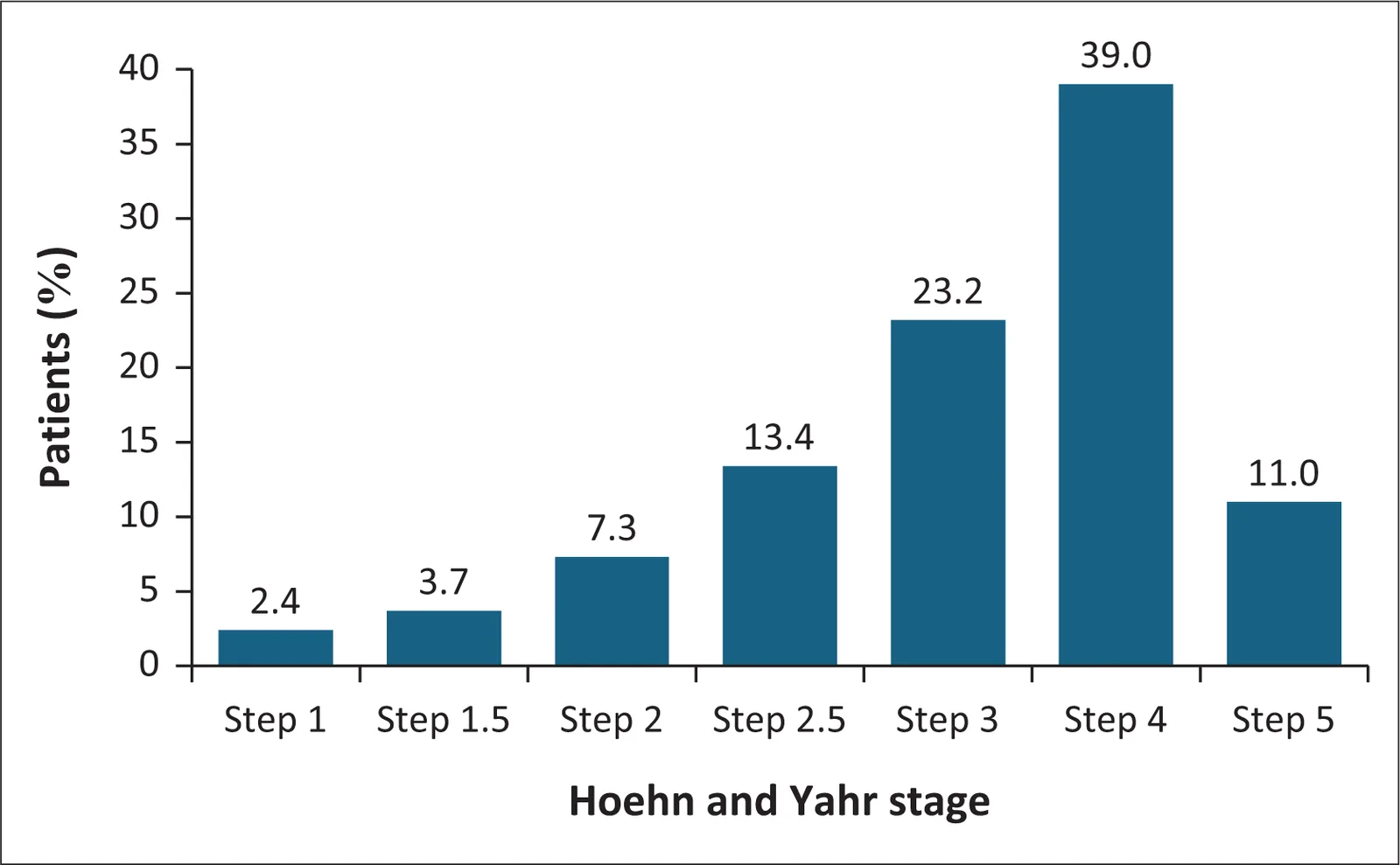
Hoehn and Yahr staging of idiopathic Parkinson’s disease severity for patients attending the movement disorder clinic at the Charlotte Maxeke Johannesburg Academic Hospital (*N* = 82).

### Associations between disease severity and demographics

Comparisons between patients with severe and non-severe IPD are presented in Table 2. The mean age was higher in the severe group (69.2 years, s.d. 10.3) than in the non-severe group (65.6 years, s.d. 12.2), although this difference was not statistically significant (*p* = 0.187). Severe disease was slightly more common among males (61.7%) than females (38.3%), but sex was not significantly associated (*p* = 0.560).

**TABLE 2:** Comparison between patients with severe disease and those without (*N* = 82).

Variable	Mild or moderate (*n* = 22)						Severe (*n* = 60)						*p*-value
	*n*	%	Mean	s.d.	Median	IQR	*n*	%	Mean	s.d.	Median	IQR	
**Age (years)**	-	-	65.6	12.2	-	-	-	-	69.2	10.3	-	-	0.187**
**Age categories (years)**	-	-	-	-	-	-	-	-	-	-	-	-	0.436***
40–49	2	9.1	-	-	-	-	3	5.0	-	-	-	-	-
50–59	6	27.2	-	-	-	-	7	11.7	-	-	-	-	-
60–69	5	22.7	-	-	-	-	16	26.7	-	-	-	-	-
70–79	7	31.8	-	-	-	-	27	45.0	-	-	-	-	-
80+	2	9.1	-	-	-	-	7	11.7	-	-	-	-	-
**Biological sex**	-	-	-	-		-	-	-	-	-	-	-	0.560
Female	10	45.5	-	-	-	-	23	38.3	-	-	-	-	-
Male	12	55.5	-	-	-	-	37	61.7	-	-	-	-	-
**Race and/or ethnicity**	-	-	-	-	-	-	-	-	-	-	-	-	0.968***
Black African people	12	54.5	-	-	-	-	31	51.7	-	-	-	-	-
White people	8	36.4	-	-	-	-	19	31.7	-	-	-	-	-
Indian people	2	9.1	-	-	-	-	8	13.3	-	-	-	-	-
Mixed race people	0	0.0	-	-	-	-	2	3.3	-	-	-	-	-
**Medication**	-	-	-	-	-	-	-	-	-	-	-	-	0.405***
Levodopa	12	54.5	-	-	-	-	23	38.3	-	-	-	-	-
Levodopa, Pramipexole	1	4.6	-	-	-	-	1	1.7	-	-	-	-	-
Levodopa, Pramipexole, Amantadine	2	9.1	-	-	-	-	5	8.3	-	-	-	-	-
Levodopa, Amantadine	7	31.8	-	-	-	-	31	51.7	-	-	-	-	-
**Duration of treatment**	-	-	-	-	2.5	1–7	-	-	-	-	3	1.5–6	0.592*

s.d., standard deviation; IQR, interquartile range.

* , Rank-sum test *p*-value;

** , *T*-test *p*-value;

*** , Fisher’s exact *p*-value.

Similarly, no significant associations were observed between disease severity and race and/or ethnicity (*p* = 0.968), treatment regimen (*p* = 0.405), or treatment duration (*p* = 0.592). These findings suggest that, within this cohort, neither demographic nor treatment-related factors were associated with disease severity.

### Age-related comparisons

Table 3 shows further subgroup analysis comparing younger patients (< 60 years, *n* = 18) with older patients (≥ 60 years, *n* = 64). Younger patients had a significantly shorter treatment duration (median 2 years vs. 3.5 years; *p* = 0.016). They also showed a trend towards less severe motor disease, with a lower median UPDRS-III score (52 vs. 62.5; *p* = 0.053), although this did not reach statistical significance.

**TABLE 3:** Comparison of demographic and clinical characteristics by age.

Variable	Age ≥ 60 years (*n* = 64)				Age < 60 years (*n* = 18)				*p*-value
	*n*	%	Median	IQR	*n*	%	Median	IQR	
**Biological sex**	-	-	-	-	-	-	-	-	0.894
Female	26	40.6	-	-	7	38.9	-	-	-
Male	38	59.4	-	-	11	61.1	-	-	-
**Race and/or ethnicity**	-	-	-	-	-	-	-	-	0.636**
Black African people	31	48.4	-	-	12	66.7	-	-	-
White people	22	34.4	-	-	5	27.8	-	-	-
Indian people	9	14.1	-	-	1	5.6	-	-	-
Mixed race people	2	3.1	-	-	0	0.0	-	-	-
**Medication**	-	-	-	-	-	-	-	-	0.468**
Levodopa	25	39.1	-	-	10	55.5	-	-	-
Levodopa, Pramipexole	2	3.1	-	-	0	0.0	-	-	-
Levodopa, Pramipexole, Amantadine	5	7.8	-	-	2	11.1	-	-	-
Levodopa, Amantadine	32	50.0	-	-	6	33.3	-	-	-
**Duration of treatment**	-	-	3.5	2–7	-	-	2	1–4	0.016*
**UPDRS score**	-	-	62.5	49.5–78.5	-	-	52	43–65	0.053*
**Severe disease**	-	-	-	-	-	-	-	-	0.073
No	14	21.9	-	-	8	44.4	-	-	-
Yes	50	78.1	-	-	10	55.6	-	-	-

Note: Comparison between patients with IPD aged < 60 years and those 60 years or older, *N* = 82.

IQR, interquartile range; IPD, idiopathic Parkinson’s disease; UPDRS, Unified Parkinson’s Disease Rating Scale.

* , Rank-sum test *p*-value;

** , *T*-test *p*-value.

Severe disease was present in 78.1% of patients aged ≥ 60 years, compared with 55.6% of those under 60 (*p* = 0.073). While this trend suggests greater disease burden among older participants, the difference was not statistically significant.

## Discussion

In this prospective cohort study, we have described the demographic and clinical profile of patients with IPD attending the movement disorder clinic at CMJAH. The key findings are that almost three-quarters of patients have severe motor disease, and that there were no significant demographic or treatment-related predictors of severity. Younger patients had shorter treatment duration and a trend towards milder disease, though this was not statistically significant. These findings indicate that, in this South African tertiary setting, patients are often diagnosed or referred at an advanced age and late disease stage, when severe disability has already developed. Only a few incidence estimates are available in HICs, and less is known about incidence in LMICs, although secondary analyses of the Global Burden of Disease study indicated a modest increase in annual percentage change in low (0.23%) and middle (0.31%) socioeconomic countries.^9^ A similar clinical profile study was conducted in Nigerian patients with IPD that provided data characterised by delayed presentation, suggesting that it may be common in developing countries.^10^

The epidemiology of IPD in HICs is well described, with peak onset typically between 65 years and 70 years and a higher prevalence among males.^11^ The current cohort reflects these international trends, with most participants being older than 60 years and men comprising nearly 60% of the sample. Onset of IPD before 40 years is rare and accounts for fewer than 5% of cases, and is usually linked to genetic variants.^12^ In such younger patients, detailed diagnostic evaluation and genetic counselling are particularly important.^13^ We did not perform genetic testing during the study period.

A striking feature of this cohort was the high frequency of advanced disease at the time of assessment. More than half of participants were classified as Hoehn and Yahr Stage 4 or 5, indicating severe disability. This contrasts with reports from HICs, where earlier diagnosis, routine screening by general practitioners, and greater public awareness often result in patients being referred and diagnosed at earlier stages.^1^ In South Africa, and LMICs more broadly, delays in diagnosis are common due to multiple factors, including low health literacy, cultural perceptions of ageing, limited access to neurologists, and long referral pathways from primary care to tertiary services.^7^ By the time patients present to specialist clinics, substantial disease progression has already occurred, which likely explains the predominance of severe disease in this study.

The treatment patterns observed also reflect resource constraints typical of LMICs. All patients in this study had been initiated on levodopa, which remains the cornerstone of therapy worldwide.^6^ However, in HICs, treatment algorithms are broader, often incorporating dopamine agonists, MAO-B inhibitors, Catechol-O-Methyltransferase (COMT) inhibitors, and advanced therapies such as DBS, apomorphine infusion, and levodopa-carbidopa intestinal gel.^1^ These options help manage motor fluctuations and improve quality of life in advanced stages. By contrast, in South Africa, access to adjuvant medications is inconsistent and device-aided therapies are unavailable in the state sector.^8^ In this cohort, levodopa and amantadine accounted for the majority of treatment regimens, while use of pramipexole was minimal. The absence of advanced therapies means that patients remain on baseline therapy despite progression, contributing to the high burden of severe disease.

The clinical implications of such late-stage presentation are substantial. Advanced motor disability is strongly associated with falls, fractures, and loss of independence. Hip fractures, in particular, are a major cause of morbidity in patients with IPD.^14^ Regular evaluation of fall risk, bone health, and fracture prevention measures should be emphasised as essential practices in South African clinics treating IPD. Furthermore, the linking of UPDRS-III scores to Hoehn and Yahr stages, as applied in this study, may provide clinicians with a practical framework for monitoring severity and guiding referral for advanced interventions where available. There is a growing burden of neurodegenerative disorders in LMICs owing to demographic changes such as population growth and ageing, and therefore reliable data on epidemiology, risk factors, preventative strategies and service delivery are needed.^15^

Treatment-related complications are mentioned in the literature. Levodopa, while highly effective, is associated with motor fluctuations due to its short half-life and variable absorption.^2^ The mechanisms of motor fluctuations associated with levodopa are poorly described, and whether these fluctuations that complicate IPD are directly related to duration of treatment or disease has not been determined.^16^ In this cohort, some participants had adjunctive therapy with amantadine or pramipexole as recorded in the medical records. Amantadine reduces dyskinesias and fluctuations, while pramipexole improves motor control and quality of life.^6^ However, in HICs, additional strategies such as DBS or infusion therapies would typically be considered in patients with severe fluctuations, underlining the inequity in available treatments between LMIC and HIC settings.

This study has several limitations. It was hospital-based and conducted at a single tertiary centre, which limits the generalisability of findings to the wider population. Patients attending specialist referral clinics are also more likely to have advanced disease, which may overestimate the prevalence and severity of IPD in the community. In addition, the cross-sectional design did not allow assessment of longitudinal progression, and non-motor manifestations of IPD were not assessed.

Despite these limitations, this study provides valuable data on the burden and characteristics of IPD in our setting. The findings reinforce the clear need for earlier recognition and quicker referral pathways to ensure that patients are diagnosed before advanced disability occurs. They also highlight the need to strengthen access to a wider range of therapies, including dopamine agonists, COMT inhibitors, and device-aided interventions, within the South African public sector. Improving representation of underrepresented populations in research is an important step towards improving access to IPD diagnosis and treatment.^17^

## Conclusion

This study demonstrates that most patients with IPD attending a tertiary referral clinic in Johannesburg have advanced motor disease. These findings underscore the importance of public health initiatives to raise awareness of early symptoms, train primary care providers in recognition and referral, and improve access to specialised movement disorder services. Expanding the availability of advanced therapies in LMICs is critical to reducing disability and improving outcomes for patients with IPD.

## References

[1] Bloem BR, Okun MS, Klein C. Parkinson’s disease. Lancet. 2021;397(10291):2284–2303. 10.1016/S0140-6736(21)00218-X 33848468

[2] Tanner CM, Ostrem JL. Parkinson’s disease. N Engl J Med. 2024;391(5):442–452. 10.1056/NEJMra2401857 39083773

[3] Dlamini WW, Nielsen S, Ushe M, Nelson G, Racette BA. A rapid motor task-based screening tool for parkinsonism in community-based studies. Front Neurol. 2021;12:653066. 10.3389/fneur.2021.653066 PMC8155367 34054697

[4] Pereira GM, Teixeira-Dos-Santos D, Soares NM, et al. A systematic review and meta-analysis of the prevalence of Parkinson’s disease in lower to upper-middle-income countries. NPJ Parkinsons Dis. 2024;10(1):181. 10.1038/s41531-024-00779-y PMC11442769 39349513

[5] Martinez-Martin P, Skorvanek M, Rojo-Abuin JM, et al. Validation study of the Hoehn and Yahr scale included in the MDS-UPDRS. Mov Disord. 2018;33(4):651–652. 10.1002/mds.27242 29322548

[6] De Bie RMA, Katzenschlager R, Swinnen BEKS, et al. Update on treatments for Parkinson’s disease motor fluctuations – An International Parkinson and Movement Disorder Society evidence-based medicine review. Mov Disord. 2025;40(5):776–794. 10.1002/mds.30162 PMC12089898 40055961

[7] Nelson G, Ndlovu N, Christofides N, Hlungwani TM, Faust I, Racette BA. Validation of Parkinson’s disease-related questionnaires in South Africa. Parkinsons Dis. 2020;2020:7542138. 10.1155/2020/7542138 PMC7306845 32617145

[8] Amod FH, Bhigjee AI. Clinical series of Parkinson’s disease in KwaZulu-Natal, South Africa: Retrospective chart review. J Neurol Sci. 2019;401:62–65. 10.1016/j.jns.2019.03.023 31015149

[9] Ben-Shlomo Y, Darweesh S, Llibre-Guerra J, Marras C, San Luciano M, Tanner C. The epidemiology of Parkinson’s disease. Lancet. 2024;403(10423):283–292. 10.1016/S0140-6736(23)01419-8 PMC11123577 38245248

[10] Okubadejo NU, Ojo OO, Oshinaike OO. Clinical profile of parkinsonism and Parkinson’s disease in Lagos, Southwestern Nigeria. BMC Neurol. 2010;10:1. 10.1186/1471-2377-10-1 PMC2806862 20051133

[11] Beitz JM. Parkinson’s disease: A review. Front Biosci. 2014;6(1):65–74. 10.2741/S415 24389262

[12] Pringsheim T, Jette N, Frolkis A, Steeves TD. The prevalence of Parkinson’s disease: A systematic review and meta-analysis. Mov Disord. 2014;29(13):1583–1590. 10.1002/mds.25945 24976103

[13] Post B, Van den Heuvel L, Van Prooije T, Van Ruissen X, Van de Warrenburg B, Nonnekes J. Young-onset Parkinson’s disease: A modern and tailored approach. J Parkinsons Dis. 2020;10(s1):S29–S36. 10.3233/JPD-202135 PMC7592661 32651336

[14] Koo HY, Cho EB, Kong SH, et al. Fracture risk in Parkinson’s disease according to its severity and duration. Osteoporos Int. 2023;34(1):81–89. 10.1007/s00198-022-06562-0 36205727

[15] Winkler AS. The growing burden of neurological disorders in low-income and middle-income countries: Priorities for policy making. Lancet Neurol. 2020;19(3):200–202. 10.1016/S1474-4422(19)30476-4 31813849

[16] Lambert D, Waters CH. Comparative tolerability of the newer generation antiparkinsonian agents. Drugs Aging. 2000;16:55–65. 10.2165/00002512-200016010-00005 10733264

[17] Sanchez AV, Ison JM, Hemley H, et al. Designing the fostering inclusivity in research engagement for underrepresented populations in Parkinson’s disease study. Contemp Clin Trials. 2022;115:106713. 10.1016/j.cct.2022.106713 35202842

